# Case report: Low dose dexmedetomidine infusion for the management of hypoglycemia in a dog with an insulinoma

**DOI:** 10.3389/fvets.2023.1161002

**Published:** 2023-04-06

**Authors:** Randolph Green, Sarah E. Musulin, Alexie Jade Baja, Bernie D. Hansen

**Affiliations:** College of Veterinary Medicine, North Carolina State University, Raleigh, NC, United States

**Keywords:** dexmedetomidine infusion, hypoglycemia, insulinoma, alpha-2 agonists, pancreatic tumor

## Abstract

**Objective:**

To describe the use of a low dose dexmedetomidine infusion as preoperative treatment for hypoglycemia secondary to a functional pancreatic tumor in a dog.

**Case summary:**

An 8.7-year-old castrated male Hungarian Vizsla presented for further evaluation of persistent hypoglycemia after the referring veterinarian established a tentative diagnosis of insulinoma based on paired insulin and glucose measurements. Abdominal ultrasound and computed tomography demonstrated evidence of a pancreatic mass with possible hepatic metastases. Attempts to aspirate the lesions under ultrasound guidance were unsuccessful, and the dog was hospitalized overnight for planned surgical resection of the presumed pancreatic tumor and biopsy of the hepatic lesions the following day. In response to a progressive increase in patient anxiety and agitation trazodone was prescribed ~5 mg/kg orally every 8 h and gabapentin at ~7 mg/kg every 8 h. As the dog continued to remain anxious dexmedetomidine at a dose of 1 mcg/kg was administered intravenously immediately followed with an infusion of dexmedetomidine at 1 mcg/kg/h. The anxious behaviors were successfully controlled with minimal cardiovascular side effects. Serial blood glucose measurements obtained during this time demonstrated euglycemia. The dog remained euglycemic while receiving dexmedetomidine for the remainder of the pre-operative period and for duration of hospitalization following surgical resection and biopsy.

**New or unique information provided:**

This case report demonstrates a possible role for dexmedetomidine to counteract hypoglycemia in dogs with insulinomas.

## Introduction

Insulinomas are insulin-secreting tumors of the pancreatic islet beta cells that are uncommon in the dog and rare in cats (1). Clinical signs are typically caused by severe hypoglycemia and can vary depending on severity. These tumors are tentatively diagnosed based on identification of persistent hypoglycemia without other explanation in animals with a normal or high plasma insulin concentration. Surgical resection yields a higher success rate than medical management practices and histopathology provides a definitive diagnosis (2). Symptomatic animals often require perioperative treatment to limit the severity of hypoglycemia and clinical signs. This is often attempted by the intravenous administration of dextrose to limit the severity of hypoglycemia. Administration of dextrose transiently corrects blood glucose concentrations however an excessive rebound release of insulin can exacerbate the hypoglycemia. Small frequent meals potentially carry similar risk as the meals may transiently increase blood glucose concentrations but result in exaggerated insulin release. Glucagon can be administered to promote glycogenolysis and gluconeogenesis however glucagon may not be readily available in many hospitals and may be cost prohibitive.

Dexmedetomidine is a potent alpha-2 agonist used widely for its sedative and analgesic properties which are accompanied by cardiovascular and neurohormonal effects. Dexmedetomidine directly inhibits insulin secretion in isolated rat pancreatic beta cells, primarily through an alpha-2 adrenoceptor, but also a GTP-binding protein pathway that eventually involves K_y_ channel activation and Ca^2+^ channel inhibition (3, 4). However, the concentrations required to produce a significant effect are higher than those expected from clinically relevant doses. Nonetheless, there is evidence of insulin suppression in animals treated with clinically relevant doses of medetomidine (dogs) or dexmedetomidine (cats). Guedes and Rude demonstrated that a single injection of medetomidine (5 mcg/kg) as a pre-anesthetic significantly reduce perioperative insulin secretion in both normal dogs and dogs with insulinoma, lessening the dose of intravenous glucose required to avoid hypoglycemia in the dogs with insulinoma (5). Bouillon et al. reported that a single large dose (10 mcg/kg) of dexmedetomidine doubled median blood glucose concentration in healthy cats from 109.01 mg/dL to 208.11 and 216.22 mg/dL (6.05 mmol/L to 11.55 and 12 mmol/L) at 1 and 2 h, respectively, with significant reduction in serum insulin concentration (6). Dexmedetomidine has also demonstrated efficacy in the reduction of serum insulin concentration secondary to the administration of glibenclamide (7). Glibenclamide is classified as a sulfonylurea, which acts by stimulating insulin release from pancreatic beta cells. Glibenclamide and other sulfonylureas are utilized in the treatment of non-insulin dependent diabetic patients. Dexmedetomidine administered at a dose of 5 mcg/kg did not alter serum glucose concentration markedly but significantly reduced serum insulin concentration in glibenclamide-induced hypoglycemia models (7). Burton et al. demonstrated the administration of medetomidine at doses of 10 and 20 mcg/kg doses transiently reduced serum insulin concentration with little effect on serum glucose concentrations in six healthy beagles (8).

This case report demonstrates the potential utility of dexmedetomidine in the pre-operative management of hypoglycemia secondary to an insulinoma. The dog described in this report demonstrated biochemical markers and histopathologic findings associated with hyperinsulinemia and the presence of an insulinoma with liver metastases.

To the authors' knowledge there are no reports describing the use of dexmedetomidine as a prolonged infusion in a hospitalized patient pre-operatively to manage hypoglycemia associated with an insulinoma. This case report demonstrates the use of a low dose dexmedetomidine infusion for the management of hypoglycemia in a dog prior to surgical resection of an insulinoma.

## Case

An 8.7-year-old Hungarian Vizsla was referred to a NC State Veterinary Hospital for further evaluation of a suspected insulinoma. The dog was initially evaluated by its primary care veterinarian in March of 2022 during a geriatric annual visit. At that time the dog was displaying an intermittent ataxic gait and bumping into objects. A complete blood cell count (CBC) and serum chemistry were performed, which demonstrated a mild hypoglycemia [63 mg/dL (3.5 mmol/L); normal reference interval not provided]. At a recheck examination 1 month later the dog's blood glucose concentration, measured twice with a handheld glucometer, was found to be 31 mg/dL (1.72 mmol/L) and 36 mg/dL (2 mmol/L). At this time blood was collected and submitted to a commercial laboratory^1^ for paired insulin and glucose assay, which demonstrated hypoglycemia [43 mg/dL (2.39 mmol/L); reference interval 70–138 (3.89–7.66)] and hyperinsulinemia (34 IU/ml; reference interval 7.5–20.0). The remainder of the serum chemistry profile was unremarkable. A tentative diagnosis of insulinoma was offered based on the elevated amended insulin:glucose ratio of 262 (9). To manage the hypoglycemia prior to referral, the dog was fed diets with a low starch content.^2^,^3^ The dog remained persistently hypoglycemic on follow up examinations with the primary veterinarian and upon presentation to NC State Veterinary Hospital.

At presentation the dog had a normal physical examination and weighed 20.8 kg. A CBC, serum chemistry and urinalysis were performed. The CBC demonstrated a reticulocytosis (106,000/ul; normal reference interval 8,040–93,730), and lymphopenia (0.541 × 10^3^/uL; 0.594–3.305). The serum chemistry demonstrated a hypoglycemia [42 mg/dL (2.33 mmol/L); (75–126) 4.16–6.99], hypophosphatemia [1.8 mg/dL; 2.6–5.3 (0.58 mmol/L; 0.84–1.71)], hypoglobulinemia [1.7 g/dL; 1.8–3.4 (17 g/L; 18–34)], hypokalemia (3.5 mmol/l; 3.6–5.3), hyperchloremia (116 mmol/l; 107–115), and a decrease in measurable bicarbonate (17 mmol/l; 18–25). Urinalysis demonstrated mild proteinuria (30 mg/dl (1+); negative) and mild bilirubinuria (2+; negative).

Computed tomography (CT) of the thorax and abdomen was performed under general anesthesia. The dog was pre-medicated with butorphanol at ~0.3 mg/kg intravenously and dexmedetomidine at ~1 mcg/kg intravenously. Prior to induction maropitant was administered at ~1 mg/kg intravenously and ondansetron at ~0.5 mg/kg intravenously. The patient was induced with propofol at 3 mg/kg and ketamine at 1 mg/kg. Prior to pre-medication the dog's blood glucose measured 51 mg/dl (2.83 mmol/L). Blood glucose concentrations were measured 30 min following induction obtaining a reading of 48 mg/dL (2.66 mmol/L). The blood glucose was measured once more at 1 h and 25 min following the initial reading, obtaining a measurement of 43 mg/dL (2.39 mmol/L). There were no other blood glucose measurements obtained during this anesthetic event and the dog was not provided any glycemic support interventions (i.e., dextrose supplementation) during this anesthetic event. The CT demonstrated normal structures in the thorax. The abdominal CT demonstrated several small round nodules of various sizes averaging 0.4 cm within the liver parenchyma. The body of the pancreas demonstrated a bulge of parenchyma that was iso-attenuating in comparison to the remainder of the pancreas pre-contrast. During the arterial phase this abnormality demonstrated strong heterogenous contrast enhancement which remained enhanced during the venous and delayed phases. The remainder of the abdominal structures were reported normal. An ultrasound guided aspirate was attempted of the patient's pancreas but aborted due to sampling difficulty reported by the radiologist.

A tentative diagnosis of an insulinoma was made based on biochemical assays and diagnostic imaging performed. The dog was hospitalized overnight for planned surgical resection of the pancreatic lesion and liver biopsies. During hospitalization the dog was administered lactated Ringer's solution^4^ at 30 mls/kg/day with potassium phosphate supplementation at a rate of 0.03 mEq/kg/h to correct for the mild hypophosphatemia and hypokalemia. In response to behavioral signs of marked separation anxiety the dog was prescribed trazodone at ~5 mg/kg every 8 h and gabapentin ~7 mg/kg every 8 h to be given orally. However, after the initial doses of these drugs the dog remained agitated with persistent vocalization, pawing at the enclosure door, panting, and pacing. Anxiolysis was then escalated to include an infusion of dexmedetomidine. An initial loading dose of 1 mcg/kg of was administered as an intravenous injection and a 7 Fr double lumen sampling catheter^5^ was placed in the right lateral saphenous vein up to the 55-centimeter mark. Once the catheter insertion was complete the infusion of dexmedetomidine was maintained at a rate of 1 mcg/kg/h. Due to recurrence of anxious behavior, the dexmedetomidine infusion rate was increased to 1.5 mcg/kg/h 10 h later, after confirmation that the behavior was not caused by recurring hypoglycemia.

At the time of catheter placement, a hand-held glucometer^6^ revealed severe hypoglycemia [20 mg/dL (1.11 mmol/L)]. Thirty minutes after the loading dose of dexmedetomidine was administered the blood glucose measured on the same device was 59 mg/dL (3.27 mmol/L), and at 1 h it increased to 106 mg/dL (5.88 mmol/L). The fluids and dexmedetomidine infusion were administered through the proximal port of the sampling catheter and blood samples for serial blood glucose measurements were obtained from the distal port. Data collection was discontinued once the dog was moved from the intermediate care unit into the anesthesia prep room for surgical resection. Serial blood glucose measurements are described in Table 1.

**Table 1:** Serial blood glucose measurements performed using the AlphaTRAK blood glucose monitoring system (see text footnote 6).

**Time (h)**	**Blood glucose measurements**
0 h	20 mg/dL (1.11 mmol/L)
0.5 h	56 mg/dL (3.27 mmol/L)
1 h	106 mg/dL (5.88 mmol/L)
3 h	75 mg/dL (4.16 mmol/L)
6 h	104 mg/dL (5.77 mmol/L)
10 h^*^	116 mg/dL (6.49 mmol/L)
11 h	68 mg/dL (3.77 mmol/L)
12 h	84 mg/dL (4.66 mmol/L)

Time 0 represents the initial blood glucose measured prior to the administration of the dexmedetomidine 1 mcg/kg bolus and initiation of the dexmedetomidine infusion. Due to the patient anxiety and the dexmedetomidine infusion was increased from 1 mcg/kg/hr to 1.5 mcg/kg/hr^*^.

The dexmedetomidine infusion was discontinued after 12 h following the initial bolus of 1 mcg/kg and the start of the infusion. This discontinuation occurred ~2 h prior to the patient moving from the intermediate care ward to the anesthesia prep room to reduce the potential for interference with intraoperative blood glucose measurements to evaluate the extent of neoplastic resection. The surgery was completed, and the dog recovered with minimal complications, remaining euglycemic through the time of discharge.

Intraoperative biopsies of the liver were taken at the time of pancreatic mass resection and yielded histopathologic diagnosis of primary pancreatic islet carcinoma with hepatic metastasis. Given the presence of a persistent hypoglycemia and concurrent hyperinsulinemia the pancreatic islet carcinoma was diagnosed as an insulinoma. During hospitalization the patient remained euglycemic while receiving the dexmedetomidine infusion and remained euglycemic to discharge. The dog was reported to be euglycemic with an increase in body weight to 23.30 kg at follow up examination 319 days post-operatively.

## Discussion

To the authors' knowledge this is the first report describing insulinoma-induced hypoglycemia in a dog that was successfully managed with a pre-operative infusion of dexmedetomidine at a clinically useful dose used to provide mild sedation. Alpha-2 agonists have been extensively utilized in veterinary medicine for their sedative and analgesic properties and are known to cause metabolic dysregulation such as suppression of insulin, suppression of renin release, enhancement of growth hormone, antidiuretic hormone release, increased gastric secretion, and decreased gastric motility.

Dexmedetomidine was administered to this dog to treat presumed separation anxiety. It is possible the behavioral changes were secondary to marked hypoglycemia, a mechanism supported by the lack of response to trazodone and gabapentin. The initial dose of 1 mcg/kg provided appropriate and effective sedation and anxiolysis allowing for insertion of a central venous catheter.

Thirty minutes following the initial dose of dexmedetomidine there was an ~3-fold increase in blood glucose concentration. With the initiation of the dexmedetomidine infusion, the dog remained euglycemic for the entirety of the 12-h infusion with the exception of hour 11, when blood glucose concentrations reached a nadir of 68 mg/dL (3.77 mmol/L). Although we did not document a change in serum insulin concentration, this finding supports the use of dexmedetomidine as a component of medical management strategy for insulinoma-induced hypoglycemia in hospitalized dogs. The dog in this case report did not receive any dextrose supplementation or glycemic drugs at any time during the administration of the infusion yet was consistently euglycemic for nearly 12 h. Measurement of the serum insulin concentration would have allowed for us to better determine whether or not the effect was associated with suppression of insulin secretion. During the anesthetic event for the CT scan the dog was administered dexmedetomidine as a premedication. There was no significant increase in blood glucose at the 30-min measurement following or the 1 h and 25-min measurement. The lack of change cannot be easily explained given the retrospective nature of this case report however it can be speculated that the time points when blood glucose was measured could represent pre-insulin suppression (30-min measurement) and the nadir (1 h and 25 min measurement). The dog received a single bolus of dexmedetomidine and not an infusion which could raise an argument that boluses in comparison to an infusion only causes a transient and not sustained effect.

Further evaluation of the effects of dexmedetomidine, particularly when administered over long periods, is needed to better understand the effects on insulin and glucose homeostasis. However, the favorable response seen in this dog suggests there may be a role for dexmedetomidine in the medical management of hospitalized dogs with persistent hypoglycemia due to hyperinsulinemia, paraneoplastic syndromes, and xylitol toxicosis.

This case report provides supporting evidence that dexmedetomidine administered in usual clinical doses for mild sedation has the potential to serve as a pre-operative management strategy for hypoglycemia in dogs with insulinoma. Measurement of serum insulin concentrations during the euglycemic periods and following the discontinuation of the infusion would further define the relationship between dexmedetomidine and glucose homeostasis.

Although this dog demonstrated a favorable response, a larger study population measuring serial insulin and glucose concentrations during the administration of a dexmedetomidine infusion should be evaluated to demonstrate a consistent and reliable association between dexmedetomidine and the potential to counteract hypoglycemia induced by insulinomas.

## Data availability statement

The original contributions presented in the study are included in the article/supplementary material, further inquiries can be directed to the corresponding author.

## Ethics statement

Ethical review and approval was not required for the animal study because this paper is a submission of a case report for a patient that was hospitalized. The patient was placed on a dexmedetomidine infusion for sedation while recognizing the potential therapeutic benefit of glycemic control based on experimental studies in rodents, dogs and humans. Written informed consent for participation was not obtained from the owners because the therapeutics provided for this dog were chosen by the medical team. The patient was very anxious and biting the cage. The patient was placed on a dexmedetomidine infusion for sedation while recognizing the potential therapeutic benefit of glycemic control. A hospital consent form was completed and signed by the client approving medical care, warranted therapies, and management practices (e.g., blood glucose monitoring in a hypoglycemic patient). Written informed consent was obtained from the participant/patient(s) for the publication of this case report.

## Author contributions

RG: substantial contributions to the conception or design of the work, acquisition, analysis, interpretation of data for the work, drafted the work and revised it critically for important intellectual content, provided approval for publication of the content, and agrees to be accountable for all aspects of the work in ensuring that questions related to the accuracy or integrity of any part of the work are appropriately investigated and resolved. SM and BH: interpretation of data for the work, revised it critically for important intellectual content, provided approval for publication of the content, and agrees to be accountable for all aspects of the work in ensuring that questions related to the accuracy or integrity of any part of the work are appropriately investigated and resolved. AB: revised manuscript critically for important intellectual content, provided approval for publication of the content, and agrees to be accountable for all aspects of the work in ensuring that questions related to the accuracy or integrity of any part of the work are appropriately investigated and resolved. All authors contributed to the article and approved the submitted version.

## Footnotes

^1^^*^Antech Diagnostic laboratories, Fountain Valley, CA.

^2^^*^Purina Pro Plan Gastroenteric Fiber, Nestle, Vevey Switzerland.

^3^^*^Royal Canin Glycobalance, MARS Iincorporated and its affiliates, McLean, VA.

^4^^*^Lactated Ringer's Solution, Abbott Laboratories, North Chicago, IL.

^5^^*^MILA 7Fr double lumen sampling catheter, MILA International Inc, Florence, KY.

^6^^*^AlphaTRAK Blood Glucose monitoring system, Zoetis, Parispanny, NJ.

## References

[1] Lunn KF Page RL. Tumors of the endocrine system. In:Vail DM Withrow SJ St Louis, editors. Small Animal Clinical Oncology. 5th ed. St. Louis, MO: Saunders (2019). p. 505–31.

[2] Polton GA White RN Brearley MJ Eastwood JM. Improved survival in a retrospective cohort of 28 dogs with insulinoma. J Small Anim Pract. (2007) 48:151–6. 10.1111/j.1748-5827.2006.00187.x 17355606

[3] Takahashi T Kawano T Eguchi S Chi H Iwata H Yokoyama M. Effects of dexmedetomidine on insulin secretion from rat pancreatic β cells. J Anesth. (2015) 29:396–402. 10.1007/s00540-014-1943-2 25376970

[4] Kodera SY Yoshida M Dezaki K Yada T Murayama T Kawakami M. Inhibition of insulin secretion from rat pancreatic islets by dexmedetomidine and medetomidine, two sedatives frequently used in clinical settings. Endocr J. (2013) 60:337–46. 10.1507/endocrj.EJ12-0308 23171706

[5] Guedes AG Rude EP. Effects of pre-operative administration of medetomidine on plasma insulin and glucose concentrations in healthy dogs and dogs with insulinoma. Vet Anaesth Analg. (2013) 40:472–81. 10.1111/vaa.12047 23714015

[6] Bouillon J Duke T Focken AP Snead EC Cosford KL. Effects of dexmedetomidine on glucose homeostasis in healthy cats. J Feline Med Surg. (2020) 22:344–9. 10.1177/1098612X19847282 31090471 PMC10814663

[7] Kallio-Kujala IJ Bennett RC Raekallio MR Yatkin E Meierjohann A Savontaus E. Effects of dexmedetomidine and MK-467 on plasma glucose, insulin and glucagon in a glibenclamide-induced canine hypoglycaemia model. Vet J. (2018) 242:33–8. 10.1016/j.tvjl.2018.09.012 30503541

[8] Burton SA Lemke KA Ihle SL Mackenzie AL. Effects of medetomidine on serum insulin and plasma glucose concentrations in clinically normal dogs. Am J Vet Res. (1997) 58:1440–2. 9401696

[9] Thompson JC Jones BR Hickson PC. The amended insulin to glucose ratio and diagnosis of insulinoma in dogs. N Z Vet J. (1995) 43:240–3. 10.1080/00480169.1995.35900 16031860

